# The harmful effects of acute PM_2.5_ exposure to the heart and a novel preventive and therapeutic function of CEOs

**DOI:** 10.1038/s41598-019-40204-6

**Published:** 2019-03-05

**Authors:** Lu Dong, Wenping Sun, Fasheng Li, Min Shi, Xianzong Meng, Chunyuan Wang, Meiling Meng, Wenqi Tang, Hui Liu, Lili Wang, Laiyu Song

**Affiliations:** 1 College of Medical Laboratory, Dalian Medical University, Dalian, 116044 Liaoning Province People’s Republic of China; 2 Department of Cardiology, Second Affiliated Hospital of Dalian Medical University, Dalian, 116023 Liaoning Province People’s Republic of China; 3 Department of Clinical Laboratory, Xinyi People’s Hospital, Xinyi, 221400 Jiangsu Province People’s Republic of China

**Keywords:** Environmental impact, Public health

## Abstract

Epidemiological researches have demonstrated the relationship between PM_2.5_ exposure and increased morbidity and mortality of cardiovascular injury. However, no effective therapeutic method was established. The purpose of this study is to investigate the effect of acute PM_2.5_ exposure on the mice heart tissue and explore the therapeutic effects of compound essential oils (CEOs) in this model. In this study, after mice were exposed to PM_2.5_ intratracheally, some obvious histopathological changes as well as some great alterations of proinflammatory cytokines were observed in the heart tissue. The imbalance of oxidative stress, the altered Ca^2+^ channel related proteins and the increased intracellular free Ca^2+^ were all involved in the heart impairment and would also be investigated in this model. The CEOs alleviated the heart impairment via its antioxidant effect rather than its anti-inflammatory function because our results revealed that oxidative stress related indicators were restored after CEOs administration. At the same time, increased concentration of intracellular free Ca^2+^ and ROS induced by PM_2.5_ were reduced after NAC (N-Acetyl-L-cysteine) administration. These data suggested that the acute PM_2.5_ exposure would damage heart tissue by inducing the inflammatory response, oxidative stress and intracellular free Ca^2+^ overload. PM_2.5_-induced oxidative stress probably increase intracellular free Ca^2+^ via RYR2 and SERCA2a. CEOs have the potential to be a novel effective and convenient therapeutic method to prevent and treat the acute heart impairment induced by PM_2.5_ via its antioxidant function.

## Introduction

PM_2.5_ (the particulate matter with an aerodynamic diameter no more than 2.5 μm) is the main air particulate pollutant in China. According to WHO’s statistics, annually, 3.7 million premature deaths are attributed to outdoor air pollution especially PM_2.5_. About 80% of those deaths are due to heart diseases and stroke^1^. Epidemiological and experimental studies have also demonstrated the causal relationship between acute PM_2.5_ exposure and elevated morbidity and mortality of cardiovascular diseases^2–5^. Many researches have been done targeting the association between PM_2.5_ and cardiovascular diseases, however, the underlying mechanism is still poorly understood. And the effective method for the treatment of heart damage caused by PM_2.5_ is urgently needed.

Oxidative stress is one of the important mechanisms of lung injury induced by acute PM_2.5_ exposure^6,7^. PM_2.5_ exposure can lead to increased levels of multiple oxidative stress markers, and further cause damage to the lung^8^. Acute PM_2.5_ exposure could induce ROS-mediated oxidative stress, which could alter the permeability of epithelial cell membrane and damage DNA resulting in cell death^9,10^. The moderate ROS production has protective effect on myocardial ischemia, but the excessive production of ROS can cause myocardial damage^11,12^. Ca^2+^ is vital for maintaining the function of cardiomyocytes, and is essential in regulating the excitation contraction coupling^13^. The imbalance of Ca^2+^ homeostasis in cardiomyocytes greatly contribute to the occurrence of various cardiovascular diseases. Intracellular Ca^2+^ overload during myocardial reperfusion can cause cardiomyocyte death and consequent cardiac dysfunction^14^. Antonella Fiordelisi *et al*. speculated that the formation of peroxides induced by PM may affect the expression of Ca^2+^ channel-associated proteins, and then increase the concentration of intracellular Ca^2+^, which eventually causes ventricular hypertrophy^15^. Traffic-related PM_2.5_ exposure caused the increase of Ca^2+^ level in immune cells, leading to the damage of immune system and many immune diseases^16^. Therefore, we suspected that the acute PM_2.5_ exposure could induce the heart damage and oxidative related mechanism might play a significant role in this model.

Essential oil (EO), containing anti-aging, anti-anxiety and anti-stress aromatic substances, is attracting more and more attentions^17–20^. Compared with EO, compound essential oils (CEOs), that contains two or more EOs, exhibiting some greater benefits for human health. Sultan MT *et al*. reported that black cumin EO was helpful in reducing the extent of myocardial and liver necrosis^21^. Syringa pinnatifolia Hems1. var. alashanensis EO has a significant protective effect against experimental myocardial ischemia. Although there are some studies focusing on the function of EOs and their effects on diseases^22^, the effects of CEOs on PM_2.5_ induced heart damage have not been studied yet.

In this study, we established an acute PM_2.5_ exposure mice model by intratracheal PM_2.5_ instillation to observe the impairment on mice hearts and investigated the underlying mechanisms. To find a potential method for the prevention and treatment of this type of heart damage and to validate the therapeutic efficacy of CEOs, mice were administrated with CEOs fumigation before PM_2.5_ exposure. Finally, the effect of CEOs on the heart impairment induced by PM_2.5_ was assessed. Besides, we also exposed mice to NAC before PM_2.5_ exposure to explore the underlying mechanisms of CEOs treatment in this model.

## Results

### Characteristics of PM_2.5_

The shape and size of the particles were examined by electron microscopy. Most of the particles are irregular and the size are less than 2.5 microns (Supp. Fig. 1). The composition of PM_2.5_ sample used in this study includes carbon, water soluble ions, metal elements, polycyclic aromatic hydrocarbons and some other trace substances. We examined the water-soluble ions, some metal elements like Ca (4.17 μmol/ml), Na (3.30 μmol/ml), Al (2.32 μmol/ml), and even some heavy metals like Zn (1.18 μmol/ml), Pb (0.12 μmol/ml) could be found in the PM_2.5_ suspension.

### An acute heart impairment induced by PM_2.5_ exposure

**Figure 1:**
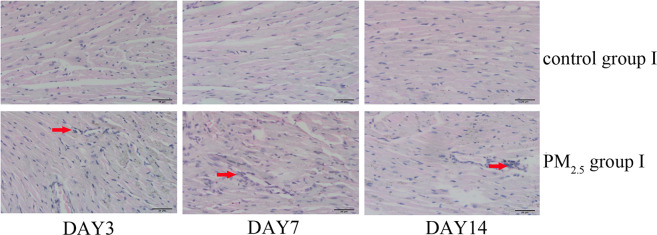
The morphological characteristics of heart tissues from saline group I and PM_2.5_ group I (400x). Mice heart tissues were obtained and samples were stained using HE. Histopathological lesions were assessed by analyses of six random fields per sample. The red arrows indicate sites of inflammatory cell infiltration. PM_2.5_ caused obvious histopathological alterations of mice heart (n = 5).

To investigate the impairment induced by PM_2.5_ in the heart tissue, mice were exposed to PM_2.5_ via intratracheal instillation. Heart tissues in control group I and PM_2.5_ group I were collected after mice were sacrificed. Morphological changes of heart tissues were observed using optical microscope after HE staining. No obvious histopathological alterations were found in control group I. However, the heart tissues of mice in PM_2.5_ group I presented a disordered arrangement of myocardial fibers, myocardial gap expansion, inflammatory cell infiltration in myocardium, and many irregularly shaped cardiocytes (Fig. 1).

### PM_2.5_ might impairs the heart tissue through inflammatory response

**Figure 2:**
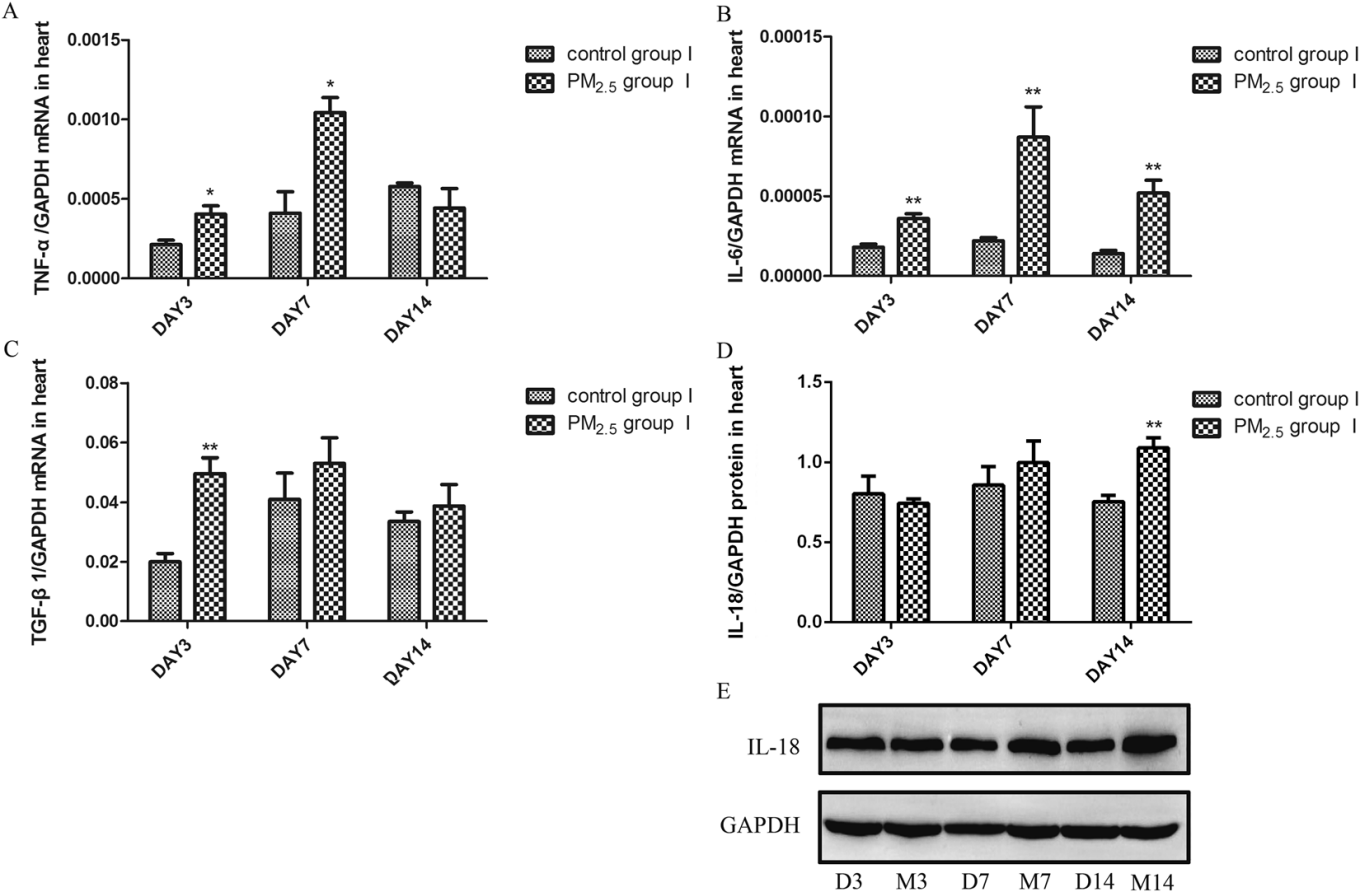
PM_2.5_ upregulated inflammatory cytokines level in heart tissues. TNF-α, IL-6 and TGF-β1 mRNA expression in heart tissues was assayed by real-time RT-PCR using the 2^−ΔCt^ method. IL-18 protein levels in heart tissues were detected by western blotting (WB). PM_2.5_ increased TNF-α (**A**) mRNA expression, IL-6 (**B**) and IL-18 (**D**,**E**) protein levels, but not altered TGF-β1 (**C**) mRNA expression. Data are expressed as mean ± standard error of the mean (n = 3–5, **P < 0.01, *P < 0.05 versus the control group I). D3, D7, D14 refers to mice in control group I sacrificed at day 3, 7, 14, respectively. M3, M7, M14 refers to mice in PM_2.5_ group I sacrificed at day 3, 7, 14, respectively.

We then examined the level of inflammatory cytokines in the heart tissue to explore the related mechanisms of PM_2.5_-induced heart damage. The mRNA expression of IL-6 (Fig. 2B) in the heart tissue was significantly increased in PM_2.5_ group I than that in control group I at all three time-points. After PM_2.5_ exposure, the mRNA expression of TNF-α (Fig. 2A) was also increased significantly at day 3 and day 7. In addition, PM_2.5_ exposure significantly increased the mRNA expression of TGF-β1 (Fig. 2C) at day 3 and the protein level of IL-18 (Fig. 2D,E) at day 14. All these results reminded us of an increased inflammatory reaction in the heart tissues after PM_2.5_ acute exposure.

### PM_2.5_ might impairs the heart tissue through oxidative stress

**Figure 3:**
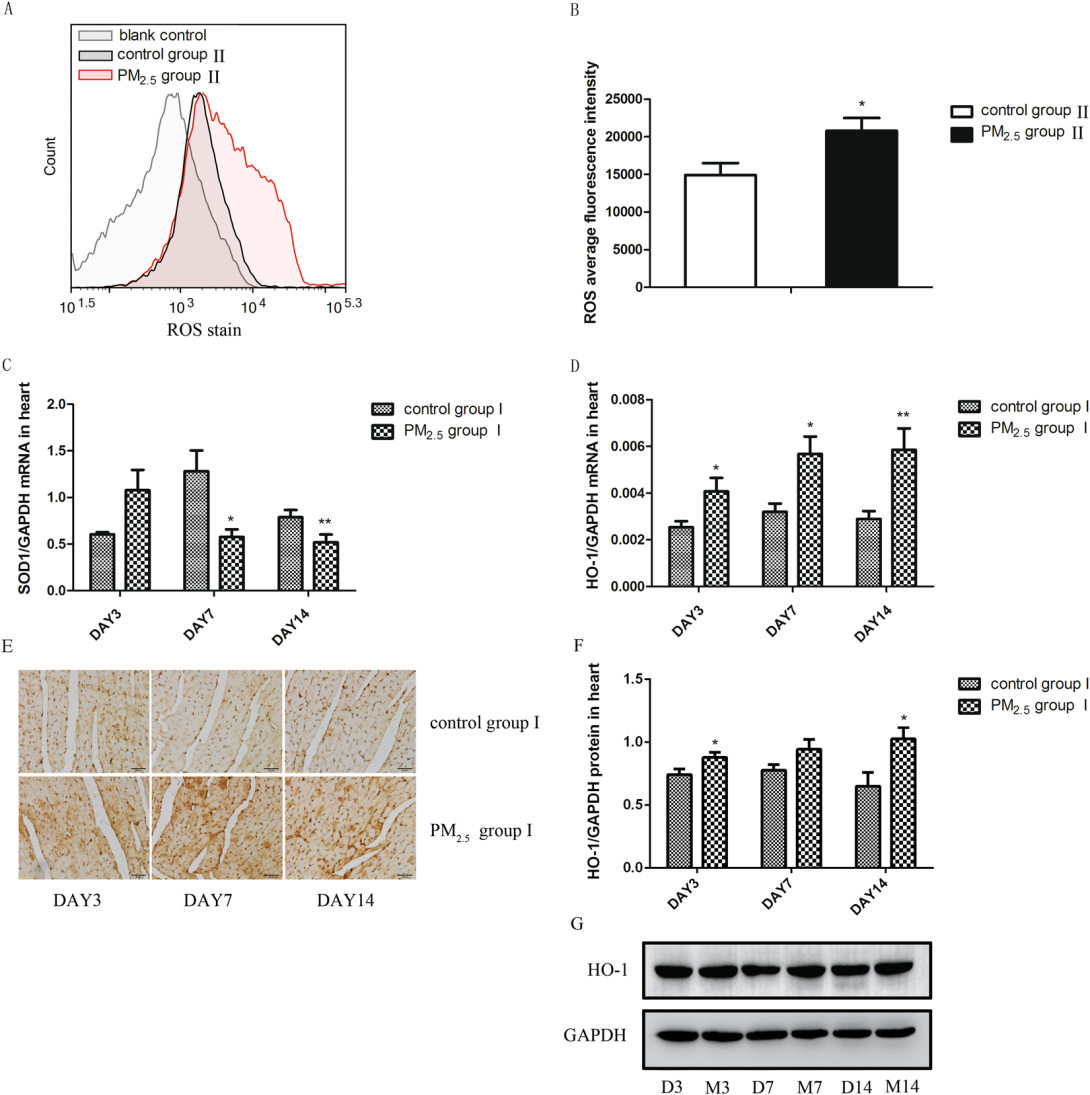
PM_2.5_ induced oxidative stress of heart tissues. The levels of ROS was determined by flow cytometry. HO-1 and SOD1 mRNA levels were assayed by real-time RT-PCR using the 2^−ΔCt^ method. HO-1 protein levels were detected by WB and IHC. PM_2.5_ increased the levels of ROS (**A**,**B**) and HO-1 (**D**–**G**) and reduced the expression of SOD1 (**C**). Data are expressed as mean ± standard error of the mean (n = 3–5, **P < 0.01, *P < 0.05 versus the control group).

Many researches suggested that oxidative stress might plays a crucial role in cardiovascular diseases. Considering the relationship between oxidative stress and PM_2.5_, we measured the level of ROS (Fig. 3A,B) in mice cardiomyocytes from control group II and PM_2.5_ group II. After PM_2.5_ exposure, the level of ROS increased obviously in the heart tissues comparing with that in control group II. HO-1 is a stress-inducible protein which is very sensitive to the oxidative stress induced by a variety of stimulus. Therefore, we detected the level of HO-1 (Fig. 3D–G) and anti-oxidant enzyme (SOD) (Fig. 3C) in the heart tissue of mice^23^. And it is notable that PM_2.5_ could induce an obvious increase of mRNA and protein expression of HO-1 at all time-points and was able to decrease the mRNA expression of SOD.

### The overload of intracellular free Ca^2+^ and the abnormal expression of Ca^2+^ channel-associated proteins in the heart tissue after PM_2.5_ exposure

**Figure 4:**
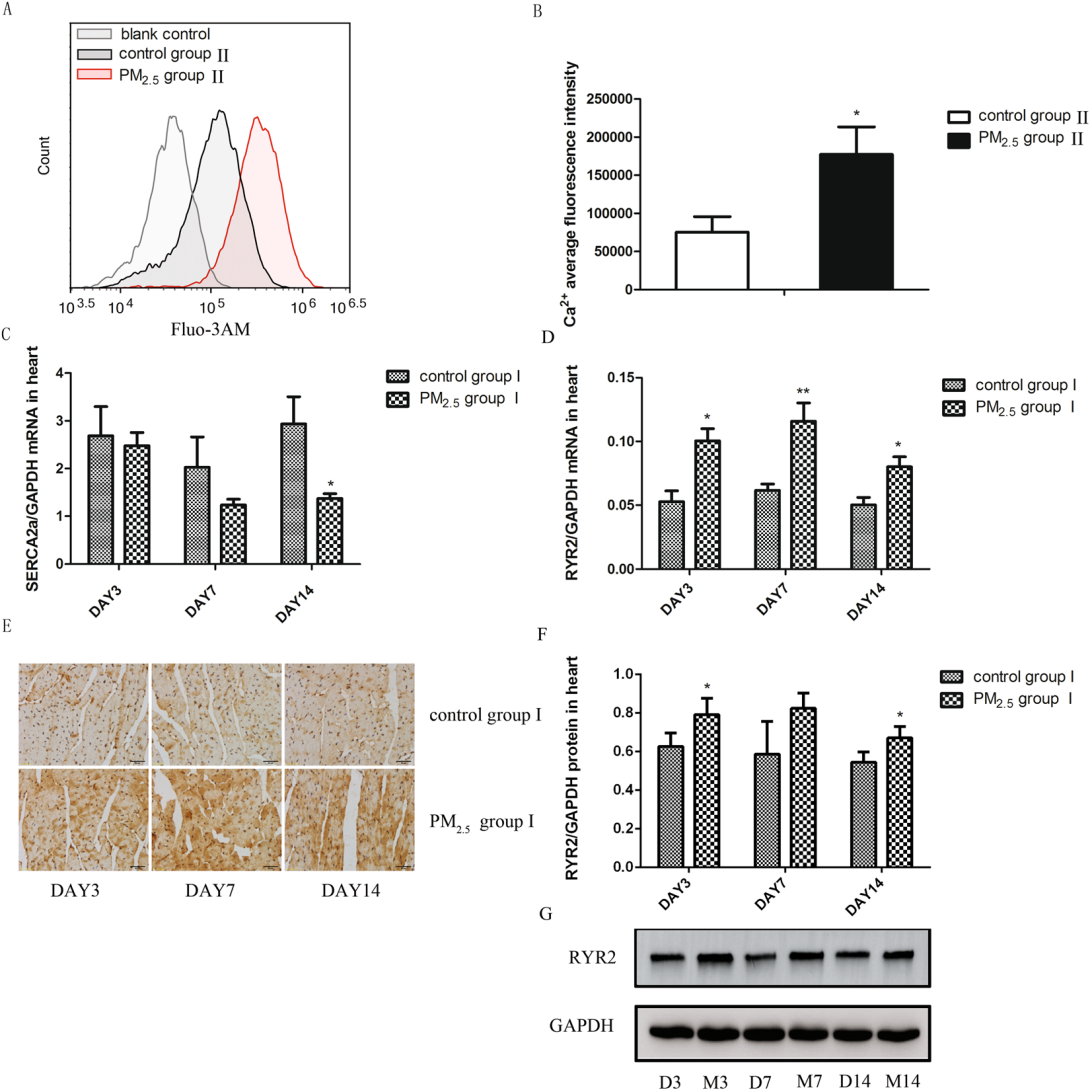
PM_2.5_ induced the overload of intracelluar Ca^2+^ and abnormal abundances of calmodulin in cardiac tissue. The level of intracellular Ca^2+^ was determined by flow cytometry. RYR2 and SERCA2a mRNA levels were assayed by real-time RT-PCR using the 2^−ΔCt^ method. RYR2 protein level was detected by WB and IHC. PM_2.5_ increased the level of intracellular Ca^2+^ (**A**,**B**) and RYR2 (**D**–**G**), and reduced the expression of SERCA2a (**C**). Data are expressed as mean ± standard error of the mean (n = 3–5, **P < 0.01, *P < 0.05 versus the control group).

Considering the pivotal role of Ca^2+^ abnormality in various cardiovascular diseases, we then examined the level of intracellular free Ca^2+^ using the Fluo3-AM in cardiomyocytes from the mice in control group II and PM_2.5_ group II. PM_2.5_ exposure significantly increased cytosolic free Ca^2+^ (Fig. 4A,B). We subsequently detected the level of Ca^2+^ channel-associated proteins. Compared with control group I, the mRNA and protein level of RYR2 (Fig. 4D–G) in the heart tissue of PM_2.5_ group I were increased obviously at all three time-points. Besides, the mRNA expression of calcium pump, SERCA2a (Fig. 4C), was decreased after PM_2.5_ exposure especially at day 14.

### CEOs may contribute to improving the heart impairment induced by PM_2.5_ exposure

**Figure 5:**
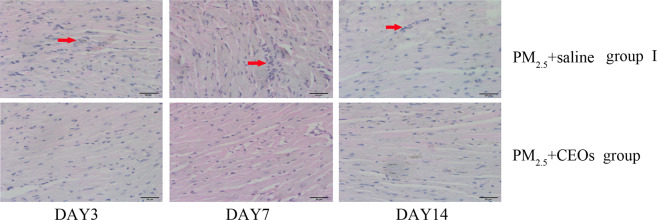
The morphological characteristics of heart tissues from PM_2.5_ + saline group I and PM_2.5_ + CEOs group (400x). Mice heart tissues were obtained at day 3, 7, 14 after administration by PM_2.5_. Samples were stained using HE. Histopathological lesions and changes were assessed by histological analyses of six random fields per sample by optical microscope. The red arrows indicate sites of inflammatory cell infiltration. CEOs improved the disorder of myocardial fibers and the infiltration of inflammatory cells induced by PM_2.5_ exposure (n = 5).

The antioxidant and anti-tumor activities of EOs have been widely confirmed by previous researches. Therefore, we used CEOs in this model to investigate whether CEOs could be a potential drug for prevention and treatment of heart damage induced by acute PM_2.5_ exposure. We exposed mice to saline alone or CEOs + saline respectively via static inhalation before PM_2.5_ exposure. After PM_2.5_ exposure, we assessed the effect of CEOs in this model. According to the histopathological results, CEOs significantly improved the disorder of myocardial fibers and reduced the PM_2.5_-induced inflammatory cells infiltration in the heart tissues (Fig. 5).

### CEOs may improve the heart impairment by suppressing PM_2.5_-induced oxidative stress and calmodulin disorder

**Figure 6:**
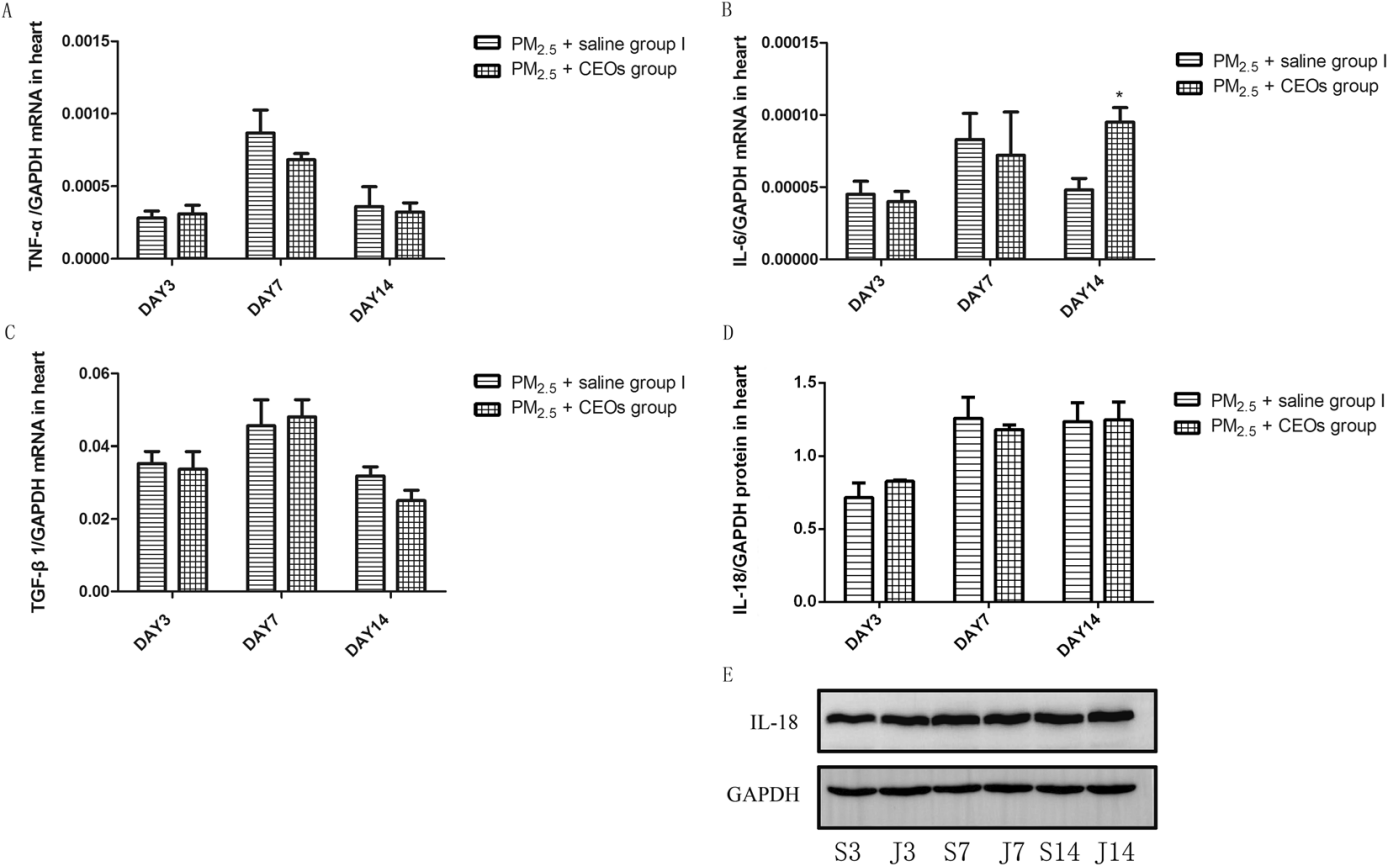
CEOs did not alter the levels of inflammatory cytokines in heart tissues. TNF-α, IL-6 and TGF-β1 mRNA expression in heart tissues was assayed by real-time RT-PCR using the 2^−ΔCt^ method. IL-18 protein level in heart tissues were detected by western blotting (WB). CEOs did not change TNF-α (**A**), IL-6 (**B**) and TGF-β1 (**C**) mRNA expression and IL-18 (**D**,**E**) protein level. Data are expressed as mean ± standard error of the mean (n = 3–5, **P < 0.01, *P < 0.05 versus the PM_2.5_ + saline group I). S3, S7, S14 refers to mice in PM_2.5_ + saline group I sacrificed at day 3, 7, 14, respectively. J3, J7, J14 refers to mice in PM_2.5_ + CEOs group sacrificed at day 3, 7, 14, respectively.

**Figure 7:**
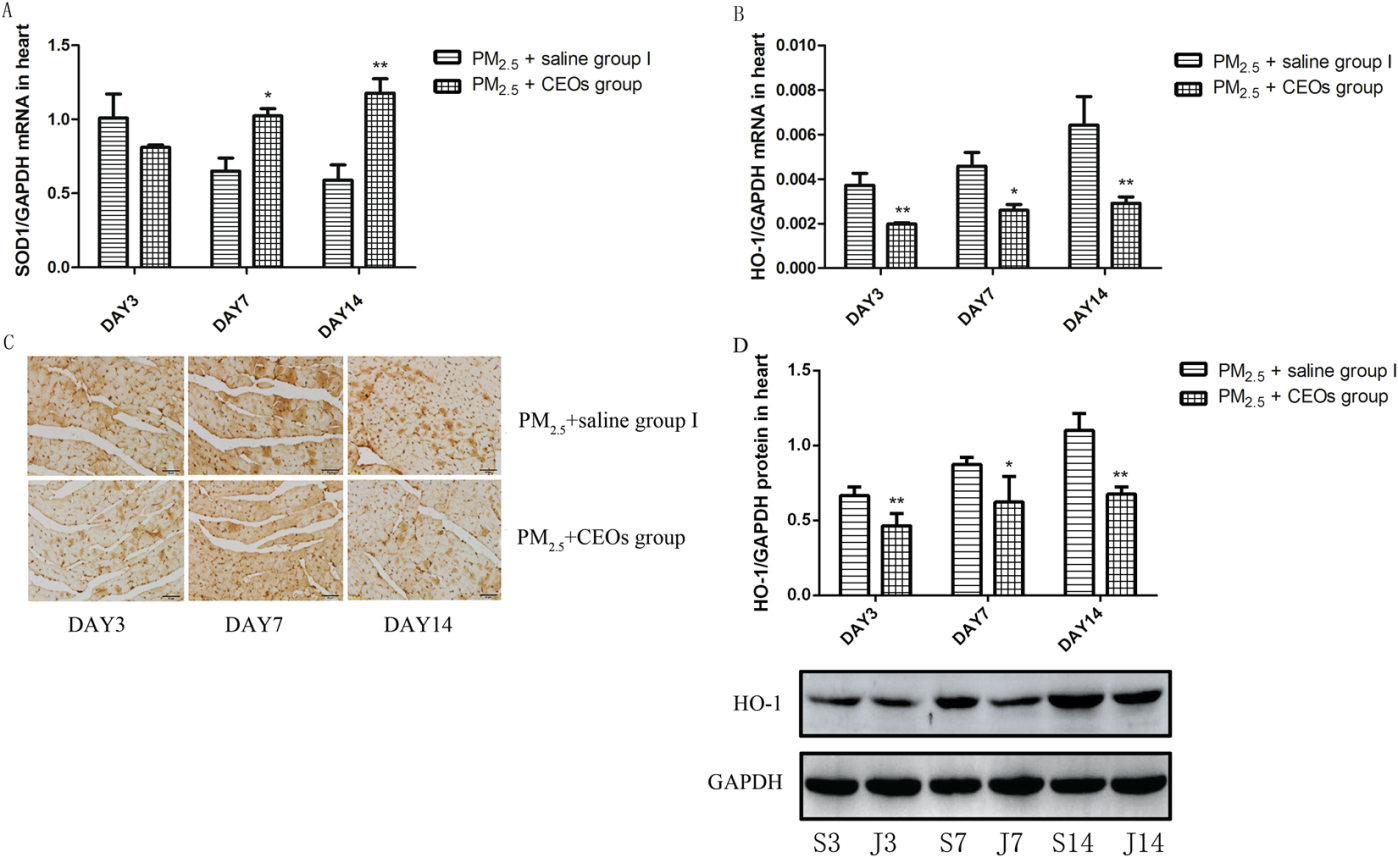
CEOs suppressed PM_2.5_-induced oxidative stress. HO-1 and SOD1 mRNA levels were assayed by real-time RT-PCR using the 2^−ΔCt^ method. HO-1 protein level was detected by WB and IHC. CEOs reduced the levels of HO-1 (**B**–**E**) and increased the expression of SOD1 (**A**). Data are expressed as mean ± standard error of the mean (n = 3–5, **P < 0.01, *P < 0.05 versus the PM_2.5_ + saline group I).

**Figure 8:**
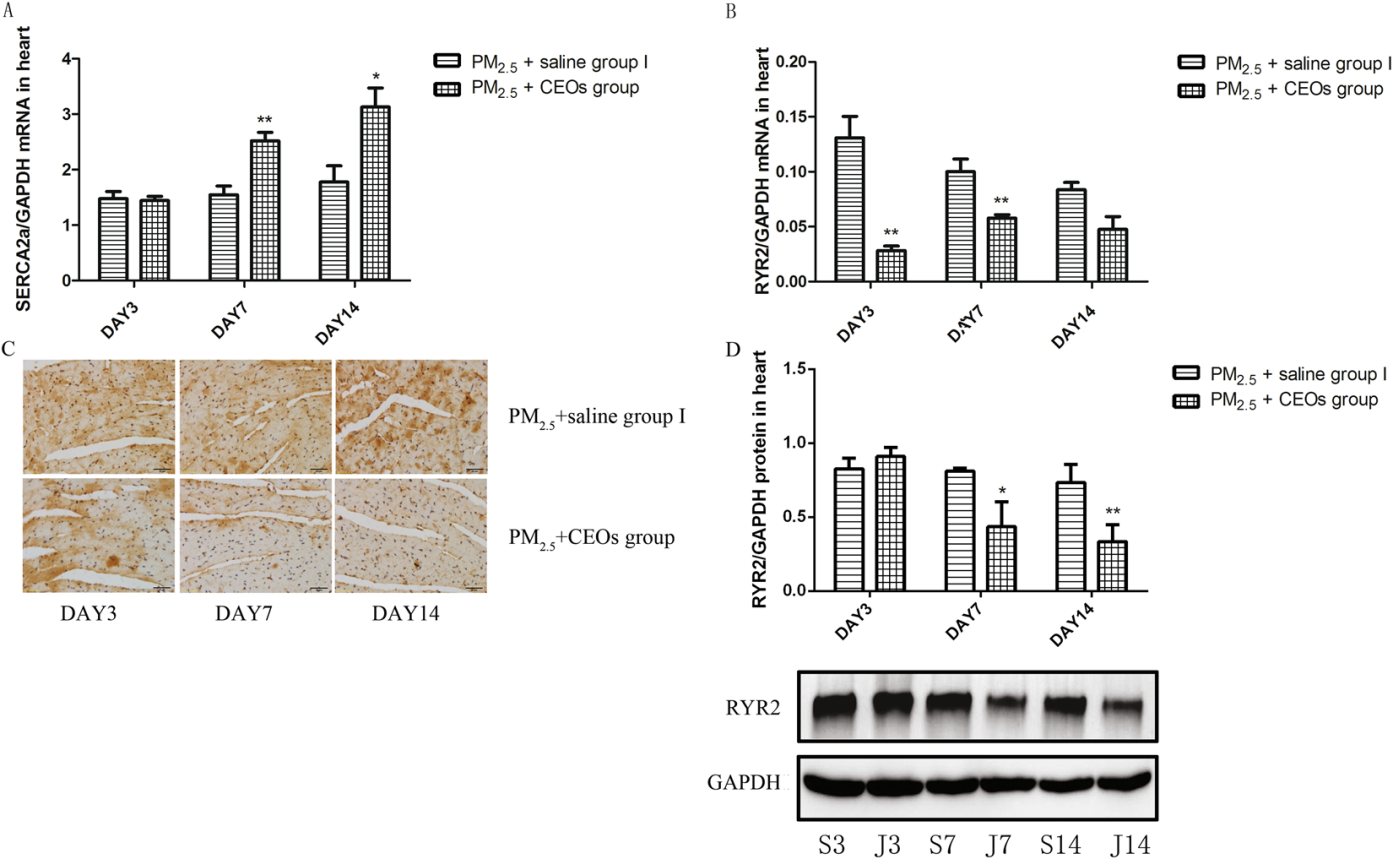
CEOs suppressed PM_2.5_-induced calmodulin disorder. RYR2 and SERCA2a mRNA levels were assayed by real-time RT-PCR using the 2^−ΔCt^ method. RYR2 protein level was detected by WB and IHC. CEOs reduced the level of RYR2 (**B**–**E**) and increased the expression of SERCA2a (**A**). Data are expressed as mean ± standard error of the mean (n = 3–5, **P < 0.01, *P < 0.05 versus the PM_2.5_ + saline group I).

To explore the potential mechanism of CEOs in improving PM2.5-induced heart impairment, we evaluated the level of inflammatory cytokines, oxidative stress markers and Ca^2+^ channel-associated proteins. Our data revealed that CEOs did not alter the expression of TNF-α (Fig. 6A) and TGF-β1 (Fig. 6C) at all three time-points, as well as the expression of IL-18 (Fig. 6D–E). Unexpectedly, the mRNA expression of IL-6 (Fig. 6B) increased significantly at day 14. These results suggested that CEOs might not down-regulate the heart impairment via influencing inflammatory response in this model. However, the levels of oxidative stress markers and Ca^2+^ channel-associated proteins were altered significantly by CEOs administration. As shown in Fig. 7, the mRNA expression and protein level of HO-1 (Fig. 7B–E) were decreased significantly at all three time-points. Meanwhile, the mRNA expression of SOD1 (Fig. 7A) showed an obvious increase at day 7 and day 14. The mRNA expression of RYR2 (Fig. 8B), a Ca^2+^ channel-associated protein, was obviously decreased at all three time-points and the protein level of RYR2 (Fig. 8C–E) was also significantly decreased at day 7 and day 14. Besides, the mRNA expression of Ca^2+^ pump, SERCA2a (Fig. 8A), was significantly increased at day 7 and day 14.

### NAC alleviate intracellular Ca^2+^ overload in hearts induced by PM_2.5_ exposure

**Figure 9:**
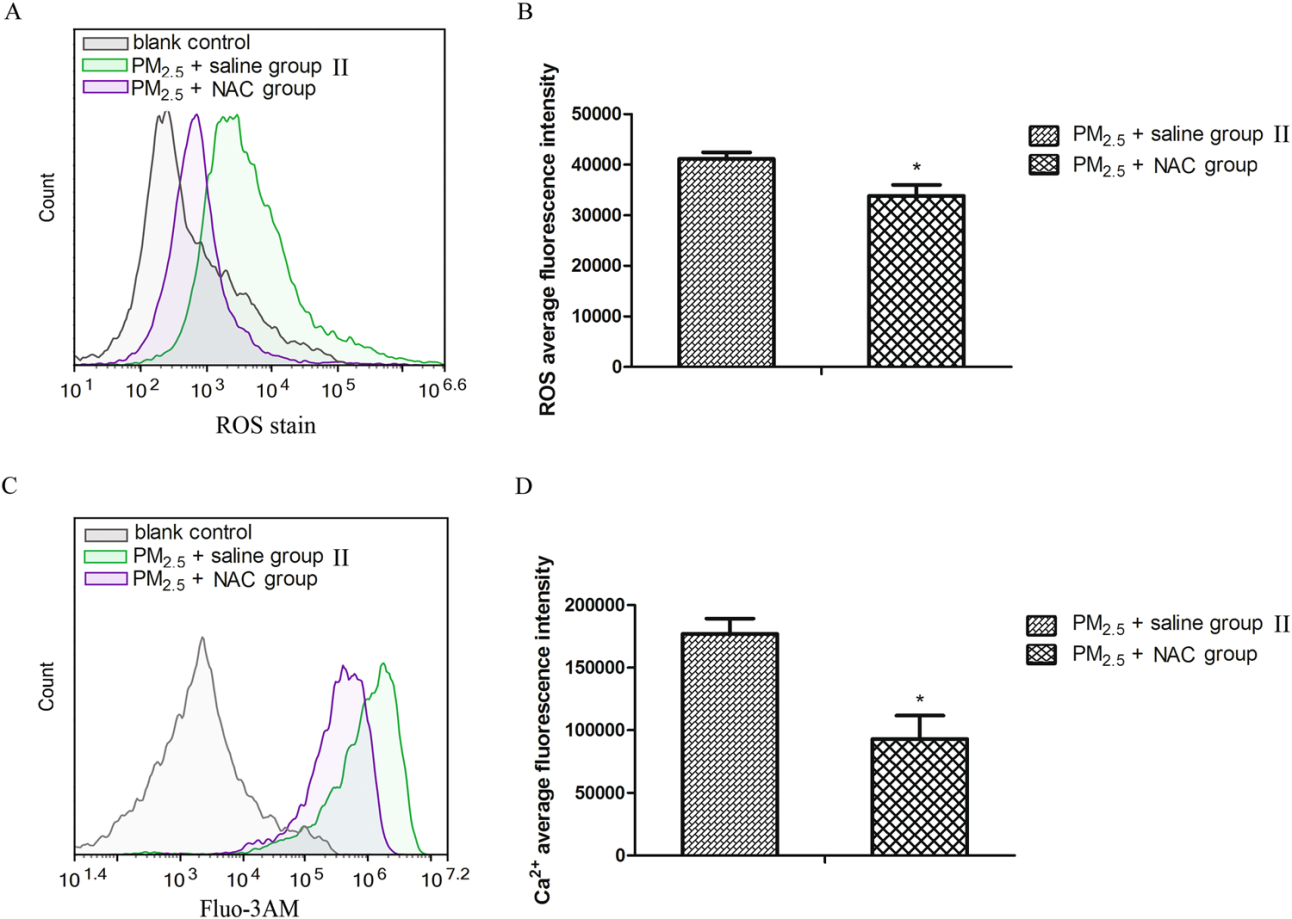
NAC alleviated increased levels of ROS and intracellular Ca^2+^ in hearts induced by PM_2.5_. The levels of ROS and intracellular Ca^2+^ was determined by flow cytometry. After NAC scavenging ROS (**A**,**B**), the level of intracellular Ca^2+^ (**C**,**D**) was significantly reduced. Data are expressed as mean ± standard error of the mean (n = 3–4, **P < 0.01, *P < 0.05 versus the control group II).

Some studies suggested the importance of oxidative stress in regulating intracellular free Ca^2+^ ^24,25^. We then used NAC to explore whether the decreased intracellular free Ca^2+^ after CEOs treatment was regulated by oxidative stress in this model. In acute PM_2.5_ exposed mice model, the ROS level (Fig. 9A,B) of cardiomyocytes after NAC treatment was decreased significantly. Meanwhile, the high level of PM_2.5_-induced intracellular free Ca^2+^ (Fig. 9C,D) in cardiomyocytes was attenuated significantly after NAC treatment.

## Discussion

With the rapid development of economy, air pollution has become an important issue threatening our life and health. Among all the annual premature death cases related with air pollution worldwide, especially the cases caused by PM_2.5_, about 80% premature deaths are due to heart diseases or stroke. Both epidemiological and experimental studies have demonstrated the crucial connection between acute PM_2.5_ exposure and increased morbidity and mortality of cardiovascular diseases^26–28^. Therefore, it is very necessary and important to investigate the mechanism of PM_2.5_-induced acute heart injury and explore some potential protective and therapeutic methods.

In this study, firstly, we explored the heart injury induced by acute PM_2.5_ exposure firstly. Then we observed therapeutic effects of CEOs in this model after PM_2.5_ exposure. We proposed that CEOs might play a therapeutic role mainly through its antioxidant functions. Based on that, we finally investigated the underlying mechanism behind this phenomenon.

We firstly exposed Balb/c mice to PM_2.5_ to evaluate heart impairment induced by PM_2.5_. Referring to the results of previous literatures and our pre-experiments, we chose day 3, 7, 14 to observe the oxidative stress and inflammatory response in this model^29–31^. After acute PM2.5 exposure, obvious histopathological alterations of the heart tissue could be observed. We then examined the level of inflammation related cytokines to investigate potential mechanisms of heart damage. TNF-α is an important proinflammatory cytokine mainly secreted by macrophages and mature cardiomyocyte^32^. In addition to the proinflammatory function, IL-6 is also a sensitive indicator for early tissue damage^33^. The increased TNF-α and IL-6 mRNA expression in the heart tissues suggested the upregulated inflammatory response after PM_2.5_ exposure. The elevated level of TGF-β1 in PM_2.5_ group I also reminded us a possible myocardial fibrosis and ventricular remodeling process in this mode. The proinflammatory cytokine IL-18 has been proved to be associated with the development of cardiovascular diseases^34,35^. The higher mRNA and protein expression of IL-18 in the heart tissues in PM_2.5_ group I confirmed the inflammatory response induced by PM_2.5_ exposure. These results suggested that the inflammatory response might be responsible for PM_2.5_-induced heart impairment in this model.

Considering the relationship between oxidative stress and PM_2.5_, we suspected that oxidative stress was another important mechanism in PM_2.5_-induced heart damage. Based on that, we detected the oxidative stress in the heart tissues after PM_2.5_ exposure. The flow cytometry results indicated that the acute PM_2.5_ exposure increased the ROS level in cardiomyocytes. As an important antioxidant enzyme, SOD1 plays a vital role in inhibiting ROS and resisting oxidative stress^36,37^. The decreased mRNA expression of antioxidant enzyme SOD1 after PM_2.5_ exposure reminded us an increased oxidative stress in this model. HO-1 is usually used as a sensitive indicator to represent the level of oxidative stress in many experiments. The increased mRNA and protein expression of HO-1 confirmed that PM_2.5_ could induce an aggravated oxidative stress in the heart tissues. These results suggested that oxidative stress might be another important mechanism of PM_2.5_-induced heart injury.

It is generally recognized that intracellular free Ca^2+^ overload will damage the structure and function of cells and mitochondria. Thus we detected the content of intracellular free Ca^2+^ in cardiomyocytes after PM_2.5_ exposure. In our study, PM_2.5_ acute exposure resulted in an increased level of intracellular free Ca^2+^ in cardiomyocytes. This result suggested the Ca^2+^ overload of cardiomyocytes may be responsible for PM_2.5_-induced heart injury in this model. The main function of RYR2 is releasing Ca^2+^ from the sarcoplasmic reticulum to the cytoplasm. The role of SERCA2a is to pump Ca^2+^ from cytoplasm into the sarcoplasmic reticulum. Xiaohong Yu *et al*. found that exposure to titanium dioxide nanoparticles inhibited the activities of Ca^2+^-ATPase, Na^+^/K^+^-ATPase and Ca^2+^/Mg^2+^-ATPase in the mouse heart^38^. Long-term exposure to PM_2.5_ has been suggested to reduce the expression of SERCA2a in the heart tissue and result in a cardiac phenotype consistent with incipient heart failure^39,40^. Therefore, we detected the expression of calmodulin, RYR2 and SERCA2a in the heart tissue to make sure whether acute PM_2.5_ exposure would increase the intracellular free Ca^2+^ through RYR2 and SERCA2a. The increased mRNA and protein expression of RYR2 and the decreased expression of SERCA2a in PM_2.5_ treated mice suggested their participation in PM_2.5_-induced free Ca^2+^ overload in cardiomyocytes. Recent study suggested that the free Ca^2+^ overload in cardiomyocytes can lead to cardiomyocyte injury, induce cardiac insufficiency and promote the development of cardiovascular diseases^41^. All these results suggested that the acute PM_2.5_ exposure might increase the risk of heart diseases and even death by altering calcium homeostasis through RYR2 and SERCA2a in this model.

Till now, people have not found any clear and effective methods to prevent or treat the heart impairment induced by PM_2.5_ yet. EOs are widely used in many areas such as managing pain^42^, treating fungal infections^43^, resisting oxidative stress, anxiety, cancer and ect^44–46^. Some EOs, and their active ingredients, have been reported to be able to improve the cardiovascular system significantly by affecting vaso-relaxation, decreasing the heart rate and exerting a hypotension activity. Because of their anti-oxidative stress, anti-inflammatory, antibacterial and anti-tumor effects, EOs are widely used in the prevention and treatment of various cardiovascular diseases^47,48^. Previous investigations about EOs have already demonstrated that the mixture containing more than one EO can evoke synergic actions to exhibit some greater benefits^49^. In present reports, the synergistic effect of different EOs determines that CEOs have a better effect. Therefore, we postulated CEOs might have the potential to play a protective role in this model and even to be a novel therapeutic strategy. In this study, four types of EOs (extracted from spruce, mint, frankincense and eucalyptus separately) with a purity of 100% were mixed together to yield designated CEOs. The major ingredients of the compound CEOs including Eucalyptol, α-Pinene, p-cymene. *et al*. have been shown to be effective in antioxidant performance^50–52^, that had been examined and reported in our previous work^53^. We pre-treated mice with saline or CEOs via static inhalation before PM_2.5_ administration. Pathological results suggested CEOs significantly restrained PM_2.5_-induced heart injury. We then investigated the underlying mechanisms. After the intervention of CEOs, there was no significant changes in the expression of inflammatory cytokines in the hearts of mice after PM_2.5_ exposure, which reminded us that the CEOs might not work through regulating inflammatory response in this model. However, the oxidative stress and calmodulin disorder induced by PM_2.5_ were significantly improved by CEOs administration. These results suggested that in PM_2.5_ acute exposure mice model, CEOs may play its therapeutic role by inhibiting oxidative stress response and regulating Ca^2+^ channel-associated proteins.

Many studies have shown that ROS can cause cardiomyocyte damage by inducing calmodulin disorder and membrane lipid peroxidation, but this effect can be reduced by antioxidants^54^. Considering the therapeutic role of CEOs in this model and the decreased oxidative stress as well as the changed Ca^2+^ channel-associated proteins after CEOs administration, we wanted to validate whether the function of CEOs in this model was mainly achieved by its anti-oxidation effect. Therefore, in the following study, mice were injected with NAC intraperitoneally to inhibit PM_2.5_-induced ROS production. Flow cytometry results suggested that NAC played an effective role in reducing the ROS level in cardiomyocytes after PM_2.5_ exposure. Subsequently, the level of intracellular free Ca^2+^ was also decreased significantly after NAC administration. These results demonstrated that the imbalance of Ca^2+^ homeostasis induced by PM_2.5_ can be alleviated by scavenging ROS. It also suggested that CEOs might improve the PM_2.5_-induced calmodulin disorder by exerting its antioxidant effect, thus reducing the imbalance of Ca^2+^ homeostasis in cardiomyocytes and regulating the heart damage caused by PM_2.5_. These results provided a new direction for us to choose the more effective and more applicable treatment modalities in the next study.

## Conclusion

In summary, we demonstrated that the acute PM_2.5_-exposure can cause heart injury in mice. The oxidative stress, inflammatory response and imbalance of Ca^2+^ homeostasis may be involved in this process. CEOs alleviated the heart impairment via its antioxidant effect rather than anti-inflammatory function. Antioxidants also attenuated the increased intracellular free Ca^2+^ and shifted the imbalanced of Ca^2+^ channel related proteins induced by PM_2.5_. CEOs might be an effective and convenient therapeutic method to prevent and treat PM_2.5_-induced acute heart impairment.

## Materials and Methods

### PM_2.5_ collection, analysis and preparation

PM_2.5_ was collected on ultra-fine quartz fiber filters (General Electric, USA) using a PM_2.5_ high volume air sampler (Thermon Anderson, USA) from Langfang (Hebei, China). The filters adhering PM_2.5_ were cut into small pieces and immersed in sterile distilled water, followed by ultrasonic sonication for 2 h. The obtained particles were treated by vacuum-freeze drying, weighed and stored at −20 °C until use. Water soluble ions were measured by ICS-2000/ICS-5000 ion chromatograph (Dionex, USA). The element composition of PM_2.5_ was detected by PE-SciexDR II inductively Coupled Plasma Mass spectromete (PerkinElmer, USA). The particles were diluted with sterile saline into PM_2.5_ suspension with a concentration of 10 mg/mL. The PM_2.5_ suspension was always sonicated and vortexed before use^55^.

### Animal and treatment

#### Animal

Male Balb/c mice aged 6–8 weeks were purchased from the Changsheng biotechnology Co., LTD. (Shenyang, China). All animals were housed under standard conditions (temperature 24 ± 1 °C and humidity 50–60%) with a 12-h light-dark cycle. Food and water were freely available. The Animal Care and Use Committee of Dalian Medical University approved the all animal experiments, which complies with the National Institutes of Health Guide for the Care and Use of Laboratory Animals.

#### Experiment 1: PM_2.5_ exposure and CEOs treatment

96 Balb/c mice were randomly divided into four groups (n = 24), as follows: control group I, PM_2.5_ group I, PM_2.5_ + CEOs group, PM_2.5_ + saline group I. In this work, we established PM_2.5_ exposed mice model according to previously published methods^56,57^. Briefly, Balb/c mice were anesthetized with an injection of 10% chloral hydrate and placed on a platform. A suspension of 0.5 mg PM_2.5_ in 50 μL sterile saline was directly administered by intra-tracheal instillation. Control mice received 50 μL sterile saline. In control group I, the mice were instilled intratracheally with 50 μL sterile saline at day 0 and day 2. In PM_2.5_ group I, the mice were instilled intratracheally with 50 μL PM_2.5_ suspension at day 0 and day 2. In PM_2.5_ + CEOs group, the mice were instilled intratracheally with 50 μL PM_2.5_ suspension at day 0 and day 2 and inhaled with 100 μL CEOs in 200 μL sterile saline for 30 min every day since the day before PM_2.5_ instillation until sacrificed. In PM_2.5_ + saline group I, the mice instilled intratracheally with 50 μL the PM_2.5_ suspension at day 0 and day 2 and inhaled with 200 μL sterile saline alone every day since the day before PM_2.5_ instillation. Exposing mice to PM_2.5_ was performed using a nonsurgical intratracheal instillation method as described previously^58^. Mice were sacrificed after intratracheal instillation at days 3, 7 and 14, respectively.

#### Experiment 2: PM_2.5_ exposure and NAC treatment

Other 48 Balb/c mice were randomly divided into four groups: control group II, PM_2.5_ group II, PM_2.5_ + NAC group, PM_2.5_ + saline group II. Mice in control group II and PM_2.5_ group II were treated with 50 μL sterile saline or 50 μL PM_2.5_ suspension at day 0 and day 2. In PM_2.5_ + NAC group, the mice were treated with 50 μL PM_2.5_ suspension at day 0 and day 2 and injected with NAC intraperitoneally one hour before PM_2.5_ exposure. The treatment of mice in PM_2.5_ + saline group II was similar to those mice in PM_2.5_ + NAC group, but the NAC was replaced by equal volume of saline.

#### Pathological examination

The heart tissues were immediately removed after mice were killed. Then, some sections were fixed in 4% paraformaldehyde, dehydrated, embedded in paraffin for the HE staining. The histopathological lesions and changes of heart tissues were observed under a light microscope. Two independent researchers randomly observed and evaluated six non-coincident microscopic fields per animal.

#### RNA extraction and reverse transcriptase-real-time quantitative polymerase chain reaction

Ventricle tissue samples were homogenized in TRIzol reagent (Invitrogen, Carlsbad, CA, USA), and total RNA was extracted from the tissues according to the manufacturer’s protocol. After removing gDNA, the total RNA (1 μg) was reversely transcribed into cDNA (RR047A; Takara, Dalian, China). Primers were designed using Primer 5, and sequences were submitted to BLAST (http://blast.ncbi.nlm.nih.gov/Blast.cgi). 2 μL of cDNA products were used in each 25 μL-PCR volume for amplification with a SYBR Premix Ex Taq II kit (RR820A; Takara, Dalian, China). Results were analyzed by using TP800 system software. GAPDH was used as loading control. The relative quantification of the expression of target gene was calculated using the 2^−ΔCt^ method^59^.

#### Immunohistochemical staining

Paraffin-embedded heart tissues were cut into 4-μm-thick sections, and deparaffinized using a graded series of xylene and ethanol. After blocking endogenous peroxidase and retrieving the antigen, the heart tissue sections were incubated with a primary rabbit anti-HO-1 (1:200, proteintech, China) and anti-RYR2 (1:400, proteintech, China) overnight at 4 °C, which were subsequently incubated with a secondary antibody at 37 °C for 30 min. Images were observed under a light microscope.

#### Western blotting

The heart total proteins were extracted and determined (KeyGEN BioTECH, Nanjing, China). After boiling, the samples containing 50 µg of proteins were separated using SDS–PAGE and wet-transferred onto PVDF membranes. The PVDF membranes were blocked for 2 h at room temperature with 5% nonfat milk, then incubated with the primary anti-RYR2 (1:800, proteintech, China), anti-HO-1 (1:600, proteintech, China), anti-IL-18 (1:500, abcam, USA) and anti-GAPDH antibodies (1:10000, abcam, USA) overnight at 4 °C. Then, incubating with a secondary HRP-conjugated antibody (1:5000, proteintech, China) was carried out for 2 h at room temperature. GAPDH was used as loading control.

#### Isolation and culture of ventricular myocytes

Mice were sacrificed at day 3 after PM_2.5_ exposure. The isolated ventricles were cut into small pieces and digested with Collagenase II (Solarbio, China). Isolated cells were filtered through a nylon filter and maintained for 45 min for sedimentation. Cardiomyocytes were mainly sedimented in the pellet and cardiac fibroblasts primarily left in the supernatant. Cardiomyocyte were washed and resuspended in PBS^60^.

#### Measurements the levels of intracellular free Ca^2+^ ion ([Ca^2+^]i) and ROS by flow cytometry

Cardiomyocytes were loaded with 5 μM Fluo3-AM (Beyotime biotechnology, shanghai, China) at 37 °C for 40 min in the dark, and loaded with 10 μM 2,7-dichlorodihydrofluorescein diacetate (DCFH-DA) (Sigma, Saint Louis, USA) for 20 min in the dark. Then the cardiomyocytes suspension was centrifuged at 1000 rpm for 10 min. The obtained precipitation was washed twice with PBS, and resuspended in PBS. The fluorescence intensity of Fluo3-AM probes was analyzed by flow cytometry using NovoExpress software (ACEA, Brussels, Belgium) with excitation at 488 nm and emission at 530 nm^61^.

#### Statistics and software

Results are presented as means ± standard error of the means (SEM). SPSS 17.0 (Chicago, IL, USA) was used for statistical analysis. Data analysis was performed by independent-samples t-test, or by Mann-Whitney rank-sum test if data was not normally distributed. P < 0.05 was defined as statistically significant.

### Ethics approval

All animal experiments were approved by the Institutional Animal Care and Use Committee at the Dalian Medical University.

## Supplementary information

Supplement Figure 1

## Data Availability

The data sets generated in the course of the current study are available from the corresponding author on reasonable request.
