# Correction: The harmful effects of acute PM2.5 exposure to the heart and a novel preventive and therapeutic function of CEOs

**DOI:** 10.1038/s41598-025-28775-z

**Published:** 2025-12-16

**Authors:** Lu Dong, Wenping Sun, Fasheng Li, Min Shi, Xianzong Meng, Chunyuan Wang, Meiling Meng, Wenqi Tang, Hui Liu, Lili Wang, Laiyu Song

**Affiliations:** 1 College of Medical Laboratory, Dalian Medical University, Liaoning Province, Dalian, 116044 People’s Republic of China; 2 Department of Cardiology, Second Affiliated Hospital of Dalian Medical University, Liaoning Province, Dalian, 116023 People’s Republic of China; 3 Department of Clinical Laboratory, Xinyi People’s Hospital, Jiangsu Province, Xinyi, 221400 People’s Republic of China

Correction to: *Scientific Reports* 10.1038/s41598-019-40204-6, published online 05 March 2019

This Article contains an error in Figure 8, where the image for the PM2.5 + CEOs (day 7) group in panel 8C was mistakenly duplicated and partially overlapped with that of the PM2.5 + CEOs (day 3) group.

The corrected Figure 8 is published below.

**Fig. 8:**
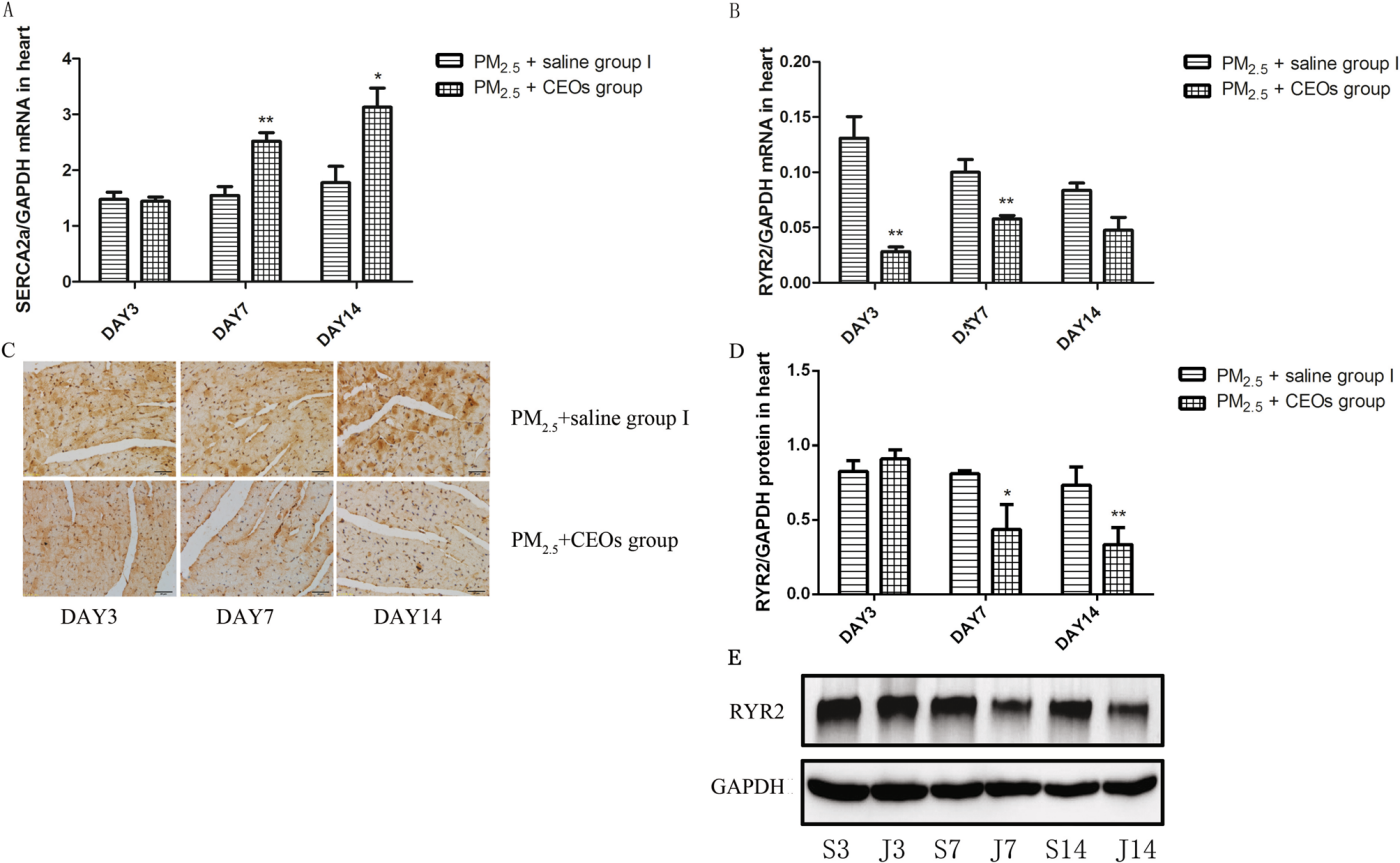
CEOs suppressed PM_2.5_-induced calmodulin disorder. RYR2 and SERCA2a mRNA levels were assayed by real-time RT-PCR using the 2^−ΔCt^ method. RYR2 protein level was detected by WB and IHC. CEOs reduced the level of RYR2 (**B**–**E**) and increased the expression of SERCA2a (**A**). Data are expressed as mean ± standard error of the mean (n = 3–5, **P < 0.01, *P < 0.05 versus the PM_2.5_ + saline group I).

