# Supplementary material for: The harmful effects of acute PM2.5 exposure to the heart and a novel preventive and therapeutic function of CEOs
Source: Sci Rep. 2019 Mar 5;9:3495. doi: 10.1038/s41598-019-40204-6 (PMC6401085; doi:10.1038/s41598-019-40204-6)
Supplement: Supplementary file 1 — Supplement Figure 1 [file 41598_2019_40204_MOESM1_ESM.pdf]

The harmful effects of acute $PM_{2.5}$ exposure to the heart and a novel preventive and therapeutic function of CEOs

Lu Dong[1,3,#], Wenping Sun[1,#], Fasheng Li[1], Min Shi[1], Xianzong Meng[1], Chunyuan Wang[1], Meiling Meng[1], Wenqi Tang[1], Hui Liu[1], Lili Wang[2,*], Laiyu Song[1,*]

[1] College of Medical Laboratory, Dalian Medical University, Dalian 116044, Liaoning Province, People's Republic of China.

[2] Department of Cardiology, Second Affiliated Hospital of Dalian Medical University, Dalian 116023, Liaoning Province, People's Republic of China.

[3]Department of Clinical Laboratory, Xinyi People's Hospital, Xinyi 221400, Jiangsu Province, People's Republic of China.

*Corresponding author: Laiyu Song (evasong1984@163.com) Tel:86-041186110388.

Lili Wang (wll_dmu@sohu.com) Tel:86-17709870768.

[#] These authors contributed equally to this work.

Supp. Fig. 1

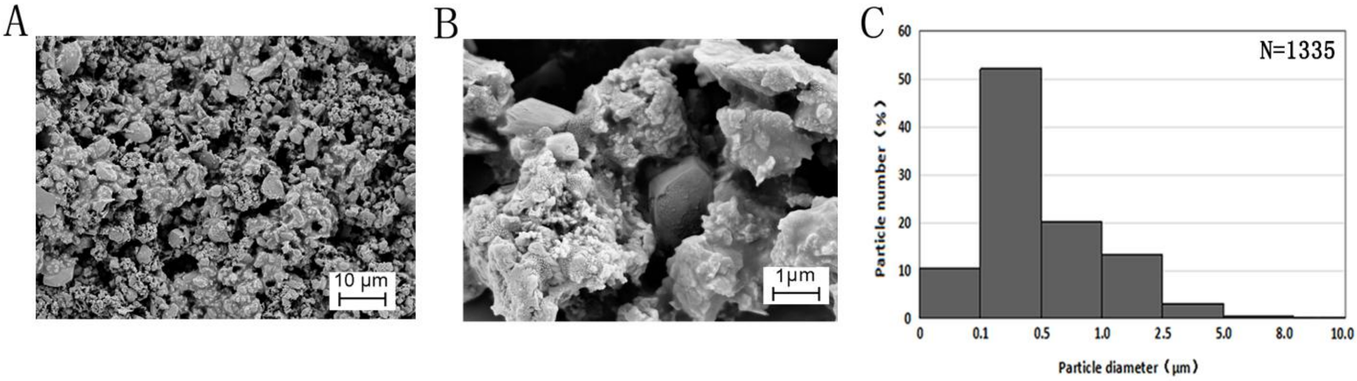

Supp. Fig.1 The shape and size of the $PM_{2.5}$ in this model. The shape and size of the particles were examined by ZEISS electron microscopy. The shape of the particles were irregular and the size distribution peaks of the particles in this model were at 0.1-0.5 μm.
